# Supplementary material for: SPIN90 Deficiency Ameliorates Amyloid β Accumulation by Regulating APP Trafficking in AD Model Mice
Source: Int J Mol Sci. 2022 Sep 12;23(18):10563. doi: 10.3390/ijms231810563 (PMC9504172; doi:10.3390/ijms231810563)
Supplement: Supplementary file 1 [file ijms-23-10563-s001.zip › Supplementary_Material_revision2_final.pdf]

Supplementary Material

1 Supplementary Data

1.1 Supplementary Figures

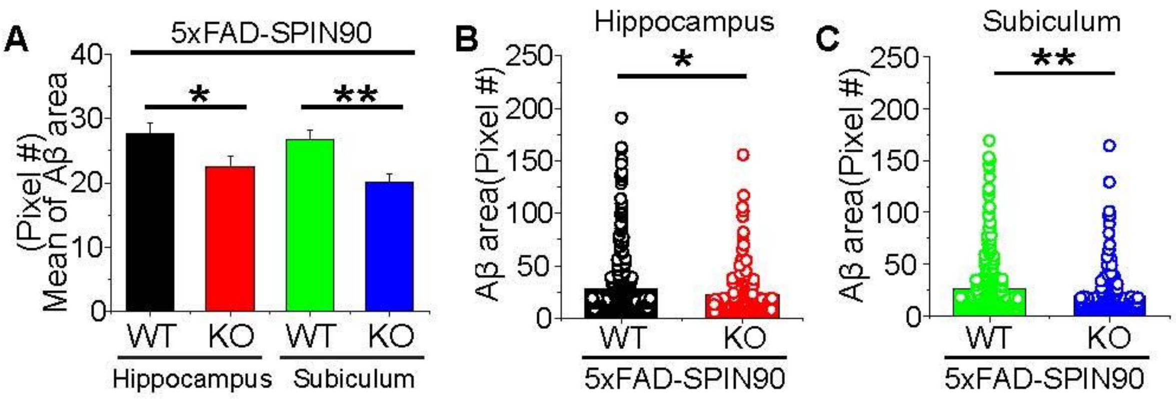

Supplementary Figure S1. Comparative sizes of areas of Aβ deposition in 5xFAD-SPIN90 WT and 5xFAD-SPIN90 KO mice. (**A**) Mean sizes of Aβ plaques in the hippocampus (27.71 ±1.52 vs. 22.44±1.70 pixels), and subiculum (26.76±1.45% vs. 20.08±1.37%) of 5xFAD-SPIN90 WT and 5xFAD-SPIN90 KO mice (n=6 each). (**B,C**) Overlay of individual Aβ plaque sizes (open circles) with mean Aβ plaque sizes in the (**B**) hippocampus and (**C**) subiculum of 5xFAD-SPIN90 WT (n=395 and n=344) and 5xFAD-SPIN90 KO (n=169 and n=237) mice. *p<0.05, **p<0.01.

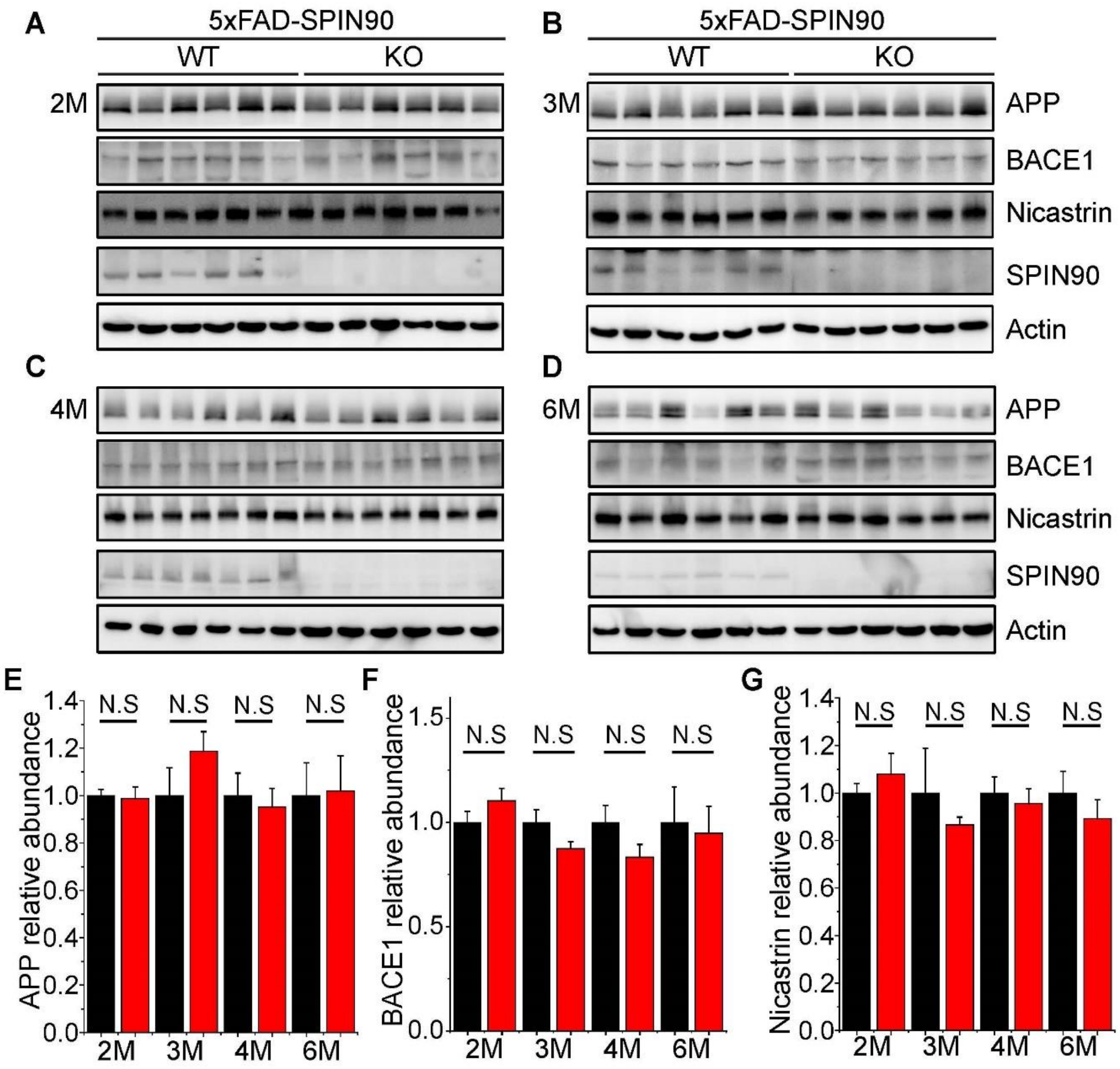

Supplementary Figure S2. Relative levels of expression of APP, BACE1, and Nicastrin in the brains of 5xFAD-SPIN90 WT and of 5xFAD-SPIN90 KO mice. (**A-D**) Representative western blots of APP, BACE1, and Nicastrin protein expression in the brains of 5xFAD-SPIN90 WT and 5xFAD-SPIN90 KO mice at **A**: 2 months; **B**: 3 months; **C**: 4 months; and **D**: 6 months. Brains were isolated from 6 mice at each time point and homogenized. Results are representative of three identical experiments. (**E-G**) Relative expression of APP (**E**), BACE1 (**F**), and Nicastrin (**G**) in 5xFAD-SPIN90 WT and 5xFAD-SPIN90 KO brains at 2, 3, 4, and 6 months. All band intensities were normalized to the intensities of actin bands in the same samples.

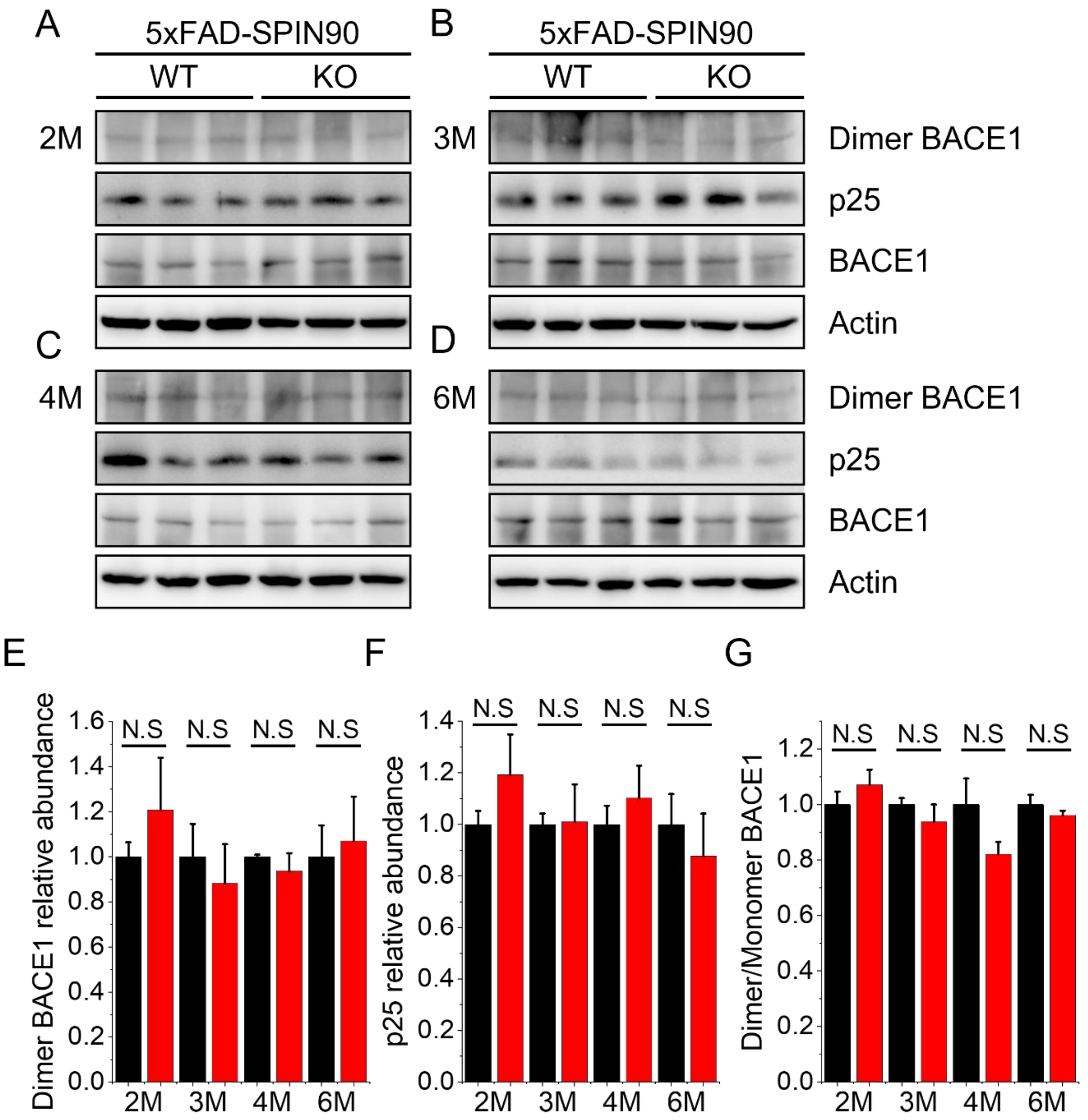

Supplementary Figure S3. The status of BACE1 protein in brains of 5xFAD-SPIN90 WT and KO mice. (**A-D**) Representative western blots of dimer BACE1, p25, and BACE1 protein expression in the brains of 5xFAD-SPIN90 WT and 5xFAD-SPIN90 KO mice at **A**: 2 months; **B**: 3 months; **C**: 4 months; and **D**: 6 months. Brains were isolated from 3 mice at each time point and homogenized. Results are representative of three identical experiments. (**E-G**) Relative expression of dimer BACE1 (**E**), p25 (**F**), and the ratio of dimer/monomer BACE1 (**G**) in 5xFAD-SPIN90 WT and 5xFAD-SPIN90 KO brains at 2, 3, 4, and 6 months. All band intensities were normalized to the intensities of actin bands in the same samples.

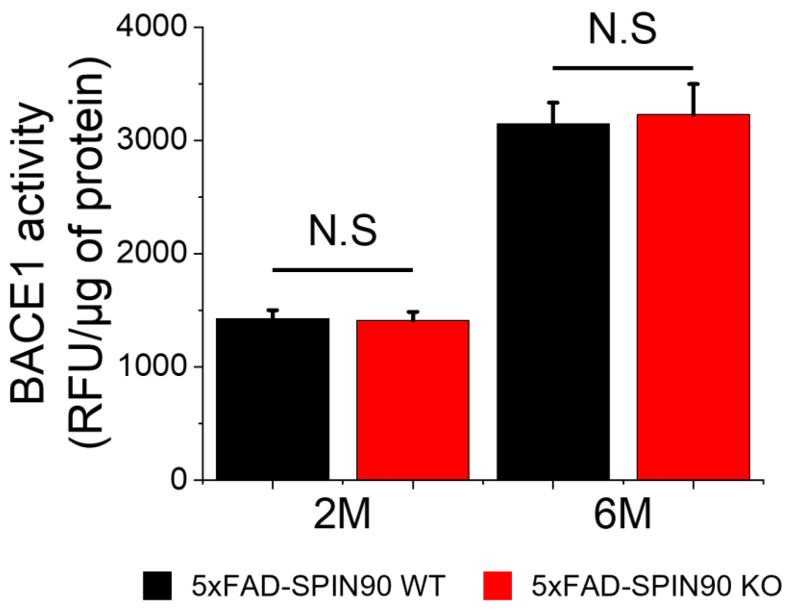

Supplementary Figure S4. BACE1 activity in the brains of 5xFAD-SPIN90 WT and KO mice. BACE1 activity in the brains of 5xFAD-SPIN90 WT and 5xFAD-SPIN90 KO mice at 2 months and 6 months was measured using β-secretase activity fluorometric assay kits. Brains were isolated from mice at corresponding time points (2 months and 6 months) and homogenized. Results are representative of three identical experiments. [2M: 5xFAD-SPIN90WT] = 1426.85±74.19 RFU/µg of protein (n=4 mice), [2M: 5xFAD-SPIN90KO] = 1410.55±74.32 RFU/µg of protein (n=4 mice), [6M: 5xFAD-SPIN90WT] = 3145.89±186.90 RFU/µg of protein (n=3 mice), [6M: 5xFAD-SPIN90KO] = 3225.21±271.05 RFU/µg of protein (n=3 mice).

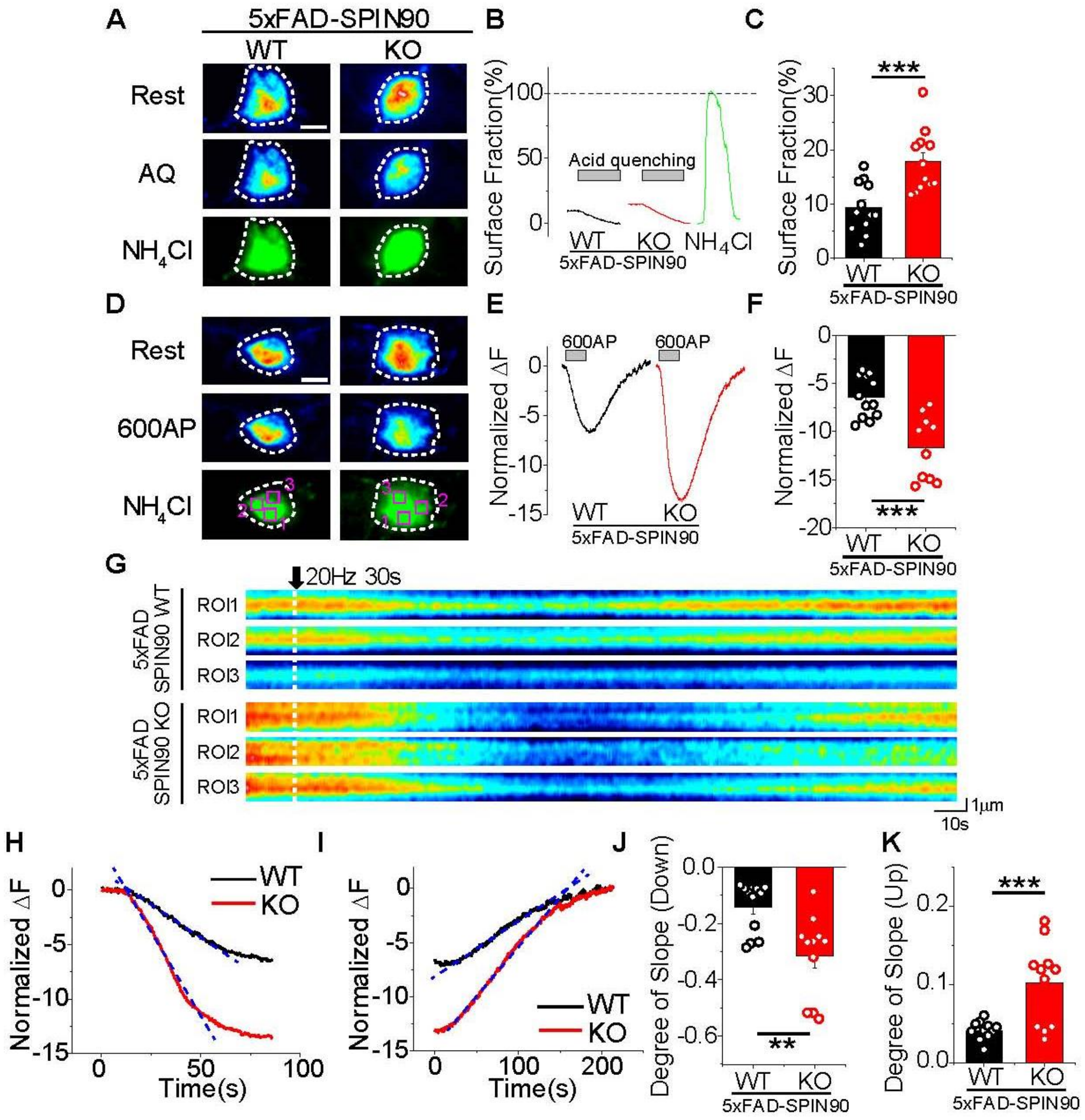

Supplementary Figure S5. Distribution and trafficking of APP in the soma is influenced by SPIN90 depletion in 5xFAD neurons. (**A**) Representative images of pH-APP in the soma of 5xFAD-SPIN90 WT (left) and 5xFAD-SPIN90 KO (right) brains at rest (top), during acid quenching (middle), and during application of NH_4Cl (bottom). Scale bar: 5 µm. (**B**) Representative traces of pH-APP in the soma in response to acid quenching in 5xFAD-SPIN90 WT (black) and 5xFAD-SPIN90 KO (red) neurons. (**C**) Mean surface fractions of pH-APP in the soma of 5xFAD-SPIN90 WT and 5xFAD-SPIN90 KO neurons. [Surface fraction: soma]$_{5xFAD\text{-}SPIN90WT}$ = 9.40±1.33 % (n=12 cells), [Surface fraction: soma]$_{5xFAD\text{-}SPIN90KO}$ = 17.85±1.66 % (n= 12 cells). (**D**) Representative images of pH-APP in the soma of 5xFAD-SPIN90 WT (left) and 5xFAD-SPIN90 (right) neurons at rest (top), after application of 600AP (middle), and after application of NH_4Cl (bottom). Scale bar: 5 µm. (**E**) Representative trace of pH-APP at axons in response to 600AP in 5xFAD-SPIN90 WT (black) and

5xFAD-SPIN90 KO (red) neurons. (**F**) Mean peak amplitudes of endocytosis in the soma of 5xFAD-SPIN90 WT and 5xFAD-SPIN90 KO neurons. $[600AP: soma]_{5xFAD\text{-}SPIN90WT}$ = -6.43±0.62 % (n=12 cells), $[600AP: soma]_{5xFAD\text{-}SPIN90KO}$ = -11.68±1.06 % (n=10 cells). (**G**) Kymographs of pH-APP in the corresponding areas of the soma in response to 600APs in 5xFAD-SPIN90 WT and 5xFAD-SPIN90 KO neurons. (**H-K**) Representative traces of pH-APP with linear fit-line during (**H**) and after (**I**) stimulation of the soma in 5xFAD-SPIN90 WT (black) and 5xFAD-SPIN90 KO (red) mice. (**J**) Mean slope of the soma, as determined by linear fitting, during stimulation of 5xFAD-SPIN90 WT and 5xFAD-SPIN90 KO neurons. $[Degree\ of\ slope: soma]_{5xFAD\text{-}SPIN90WT}$ = -0.14±0.026 (n=12 cells); $[Degree\ of\ slope: soma]_{5xFAD\text{-}SPIN90KO}$ = -0.31±0.045 (n=11 cells). (**K)** Mean slope of the soma, as determined by linear fitting, after stimulation of 5xFAD-SPIN90 WT and 5xFAD-SPIN90 KO neurons. $[Degree\ of\ slope: soma]_{5xFAD\text{-}SPIN90WT}$ = 0.04±0.004 (n=12 cells); $[Degree\ of\ slope: soma]_{5xFAD\text{-}SPIN90KO}$ = 0.10±0.02 (n=11 cells). **p<0.005, ***p<0.001.

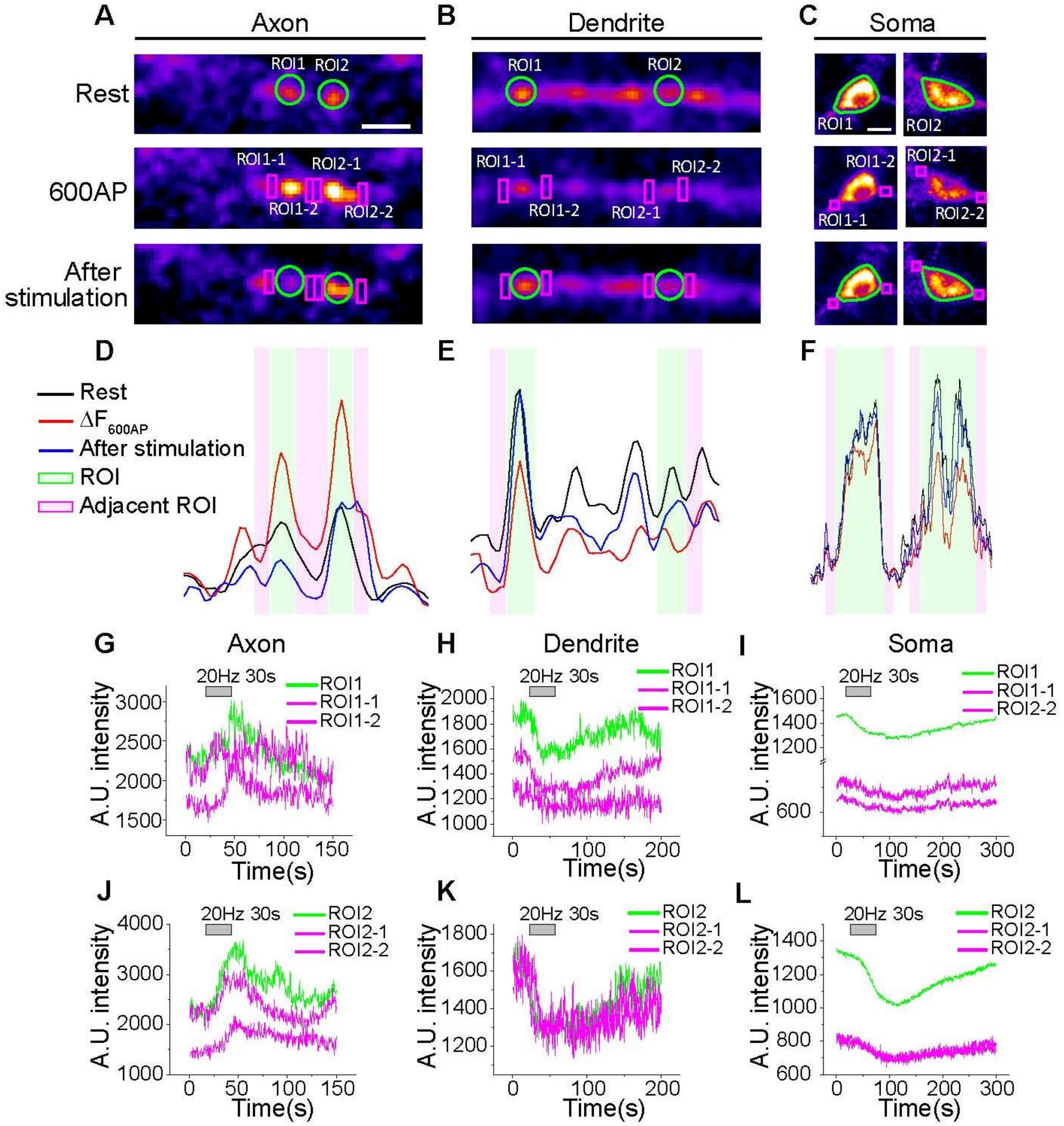

Supplementary Figure S6. Activity-dependent recycling of pH-APP between the endosomes and the surface. (**A-C**) Representative images of pH-APP at axons (**A**), dendrites (**B**), and soma (**C**) in 5xFAD-SPIN90 KO neurons at rest (top), at the peak of 600AP stimulation (middle) and at the end of stimulation (bottom). Green circles indicate regions of interest (ROI); pink squares indicate regions adjacent to ROIs. (**D-F**) Line scan profile from figure (**A-C**). (**G-L**) Fluorescence traces of pH-APP in response to 600AP of ROIs in axons (**G,J**), dendrites (**H,K**), and soma (**I,L**).

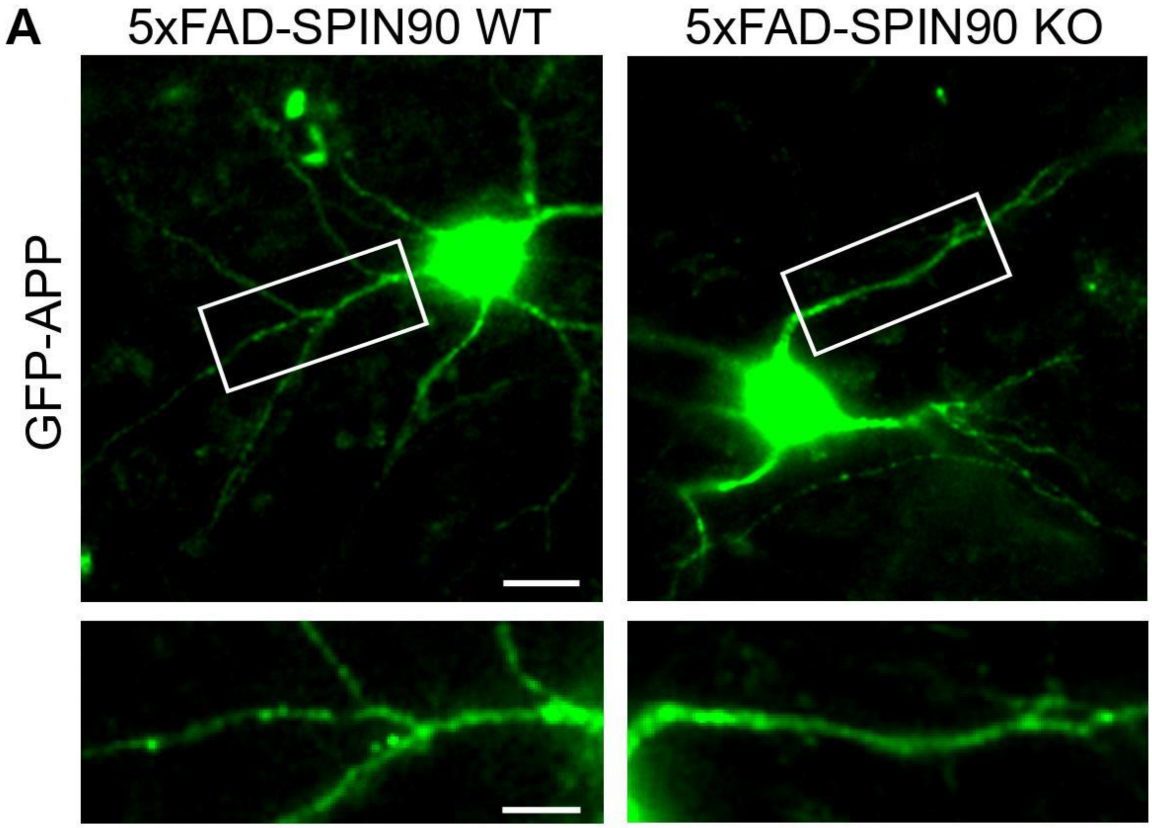

Supplementary Figure S7. APP is not accumulated in dendrites. (**A**) Representative images of GFP-APP at dendrites. Scale bar: 10 μm(top), 5 μm(bottom).

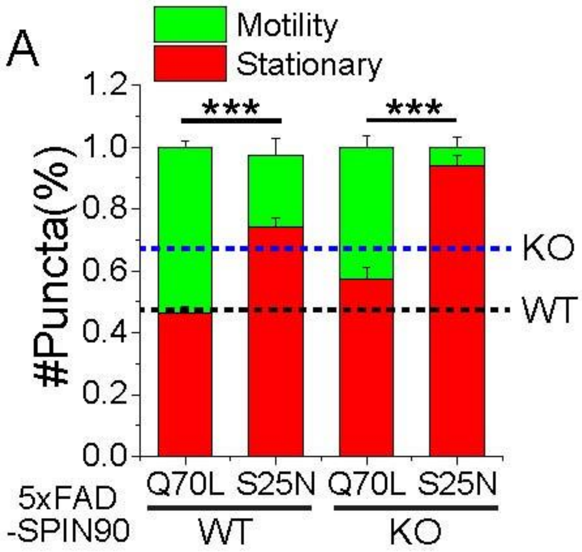

Supplementary Figure S8. Rab11 participates in axonal trafficking of APP. (**A**) Quantification of normalized mean stationary and mobile APP in 5xFAD-SPIN90 WT and 5xFAD-SPIN90 KO neurons in the presence of Rab11-Q70L or S25N. [stationary APP]$_{\text{5xFAD-SPIN90WT with Rab11A-Q70L}}$ = 0.47±0.02 (n=3 cells); [mobile APP]$_{\text{5xFAD-SPIN90WT with Rab11A-Q70L}}$ = 0.53±0.02 (n=3 cells); [stationary APP]$_{\text{5xFAD-SPIN90WT with Rab11A-S25N}}$= 0.74±0.03 (n=6 cells); [mobile APP]$_{\text{5xFAD-SPIN90WT with Rab11A-S25N}}$ = 0.24±0.05 (n=6 cells); [stationary APP]$_{\text{5xFAD-SPIN90KO with Rab11A-Q70L}}$ = 0.57±0.04 (n=7 cells); [mobile APP]$_{\text{5xFAD-SPIN90KO with Rab11A-Q70L}}$ = 0.43±0.04 (n=7 cells); [stationary APP]$_{\text{5xFAD-SPIN90KO with Rab11A-S25N}}$ = 0.94±0.03 (n=5 cells); [mobile APP]$_{\text{5xFAD-SPIN90KO with Rab11A-S25N}}$ = 0.06±0.03 (n=5 cells). ***p<0.001.
